# Electrophile-Induced Conformational Switch of the Human TRPA1 Ion Channel Detected by Mass Spectrometry

**DOI:** 10.3390/ijms21186667

**Published:** 2020-09-11

**Authors:** Lavanya Moparthi, Sven Kjellström, Per Kjellbom, Milos R. Filipovic, Peter M. Zygmunt, Urban Johanson

**Affiliations:** 1 Wallenberg Centre for Molecular Medicine (WCMM), Linköping University, SE-581 85 Linköping, Sweden; lavanya.moparthi@liu.se; 2 Department of Biomedical and Clinical Sciences (BKV), Faculty of Health Sciences, Linköping University, SE-581 85 Linköping, Sweden; 3 Division of Biochemistry and Structural Biology, Center for Molecular Protein Science, Lund University, PO Box 124, SE-221 00 Lund, Sweden; per.kjellbom@biochemistry.lu.se; 4 Division of Mass Spectrometry, Department of Clinical Sciences, Lund University, SE-22184 Lund, Sweden; sven.kjellstrom@med.lu.se; 5 Department of Chemistry and Pharmacy, Friedrich-Alexander University Erlangen-Nuremberg, Egerlandstrasse 1, 91058 Erlangen, Germany; milos.filipovic@ibgc.cnrs.fr; 6 IBGC, UMR 5095, Universite de Bordeaux, 1, rue Camille Saint Saëns, CS 61390, 33077 Bordeaux CEDEX, France; 7 Department of Clinical Sciences Malmö, Lund University, SE-214 28 Malmö, Sweden

**Keywords:** TRP channel, TRPA1, wasabi receptor, electrophile sensing, *N*-methylmaleimide, iodoacetamide, alkylated thiols, redox sensitivity, pain, mass spectrometry

## Abstract

The human Transient Receptor Potential A1 (hTRPA1) ion channel, also known as the wasabi receptor, acts as a biosensor of various potentially harmful stimuli. It is activated by a wide range of chemicals, including the electrophilic compound N-methylmaleimide (NMM), but the mechanism of activation is not fully understood. Here, we used mass spectrometry to map and quantify the covalent labeling in hTRPA1 at three different concentrations of NMM. A functional truncated version of hTRPA1 (Δ1-688 hTRPA1), lacking the large N-terminal ankyrin repeat domain (ARD), was also assessed in the same way. In the full length hTRPA1, the labeling of different cysteines ranged from nil up to 95% already at the lowest concentration of NMM, suggesting large differences in reactivity of the thiols. Most important, the labeling of some cysteine residues increased while others decreased with the concentration of NMM, both in the full length and the truncated protein. These findings indicate a conformational switch of the proteins, possibly associated with activation or desensitization of the ion channel. In addition, several lysines in the transmembrane domain and the proximal N-terminal region were labeled by NMM, raising the possibility that lysines are also key targets for electrophilic activation of hTRPA1.

## 1. Introduction

Animals have evolved a variety of biosensors to detect and avoid harmful conditions like detrimental chemicals and temperatures. Members of the transient receptor potential (TRP) ion channel family play an important role in the detection of such conditions. Among these ion channels, the sole mammalian member of the TRP ankyrin (TRPA) subfamily, transient receptor potential A1 (TRPA1), has an important function in chemical nociception. This polymodal receptor is expressed in the peripheral sensory nerve endings that originate from the dorsal root, trigeminal, and nodosa ganglia [1,2]. Similar to other TRPs, the functional unit of TRPA1 is a homotetramer, where each subunit has six transmembrane α-helices, whereof the last two and the intervening re-entrant pore loop contribute to the formation of the ion permeation pathway. Apart from the transmembrane domain, there are substantial cytosolic N- and C-terminal domains, which constitute around 80% of the mass of the TRPA1 protein [3]. The TRPA1 channel is activated by a large variety of chemicals, from exogenous plant-derived chemicals and environmental toxic reagents to endogenous inflammatory mediators, such as isothiocyanates (mustard and wasabi), cinnamaldehyde (cinnamon), allicin and diallyl disulphide (garlic), acrolein (smoke and chemotherapeutic metabolite) and nitroxyl [1,2]. To this date, more than 100 compounds including non-electrophilic compounds have been identified as TRPA1 activators and the number is still growing [1,2]. Studies have shown that such chemicals interact with TRPA1 by different mechanisms, e.g., covalent interaction of electrophilic compounds with free thiols of cysteine residues, non-covalent interaction and oxidation of cysteines that eventually promote the formation of disulphide bonds [4,5,6,7,8,9].

The human TRPA1 (hTRPA1) contains 28 cysteine, 76 lysine and 43 histidine residues, all of which are potential targets for an electrophilic attack. Studies have suggested that several conserved N-terminal cysteines in hTRPA1 (Cys421, Cys621, Cys641, and Cys665; hTRPA1 numbering will be used throughout this paper) are critical for the activation by thiol-reactive electrophiles and oxidants [5,6,10,11,12]. In addition, a lysine (Lys710) has also been shown to contribute to activation of hTRPA1 by reaction with thiocyanates and ketoaldehydes [5,13]. Several of these cysteines, as well as Lys710 are located in the pre-S1 region between the N-terminal ankyrin repeat domain (ARD) and the transmembrane domain [3].

Recently our data from single channel recordings demonstrated that activation of purified hTRPA1 and *Anopheles gambiae* TRPA1 (AgTRPA1) by electrophilic compounds occurs even in the absence of all, except Cys703 in hTRPA1, intracellular N-terminal cysteines [9,14]. In line with our findings, other groups have shown that some electrophiles, including *p*-benzoquinone, polygodial, isovalleral and (E)-2 alkenals, robustly activated heterologously expressed TRPA1 lacking three of the N-terminal critical cysteines (Cys621, Cys641 and Cys665) [15,16,17]. Knowing the reactivity of each cysteine in hTRPA1 to electrophilic compounds is crucial for understanding their biological role and may facilitate the development of new therapeutics for relief of pain. Because of species-dependent differences in response of TRPA1 upon activation by different stimuli [18] we have focused on hTRPA1 in this study. The compound N-methylmaleimide (NMM) reacts with cysteine and lysine and is a valuable tool to study electrophile TRPA1 activation [5,9,19,20,21]. Here, labeling at different concentrations of NMM was used to identify and grade accessible cysteines and lysines by mass spectrometry, which may also reveal agonist-induced structural changes of hTRPA1 similar to what has been reported for mouse TRPA1 (mTRPA1) [20,21]. In order to study the agonist binding sites outside the N-terminal ARD, the same approach was also employed on hTRPA1 without the N-terminal ARD and part of the pre-S1 region (Δ1-688 hTRPA1), forming functional tetramers that are activated by electrophilic compounds including NMM [9].

## 2. Results

**Figure 1:**
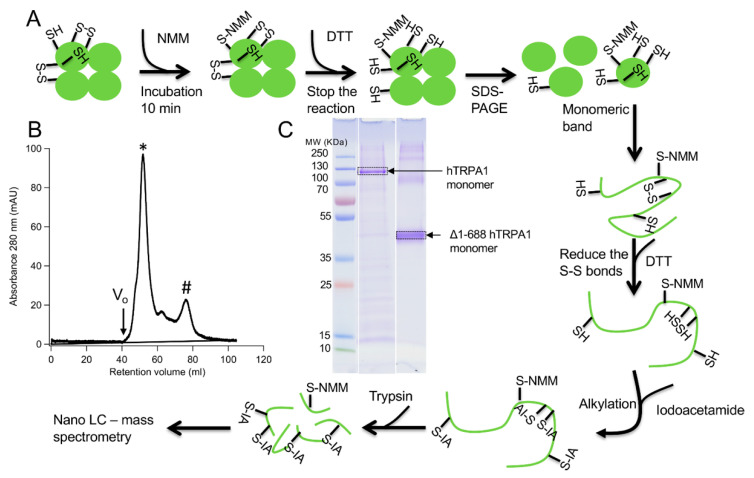
Schematic illustration of steps in the preparation of human transient receptor potential A1 (hTRPA1) proteins for the mass spectrometry analysis. (**A**) The fate of different types of cysteine residues is shown, including modification of free thiols with N-methylmaleimide (NMM), denaturation, reduction of disulphide bonds with dithiothreitol (DTT), iodoacetamide (IA) alkylation of thiols not modified by NMM, and enzymatic digestion. (**B**) Size exclusion chromatogram of the hTRPA1 sample used in the NMM labeling shows that it mainly eluted in a peak corresponding to the tetramer (*) and only a small fraction eluted as a monomer (#). V_o_ indicate void volume. (**C**) Coomassie stained SDS polyacrylamide gel of hTRPA1 and Δ1-688 hTRPA1. Marked bands are corresponding to monomers of hTRPA1 proteins and were excised from the gel for further reduction and labeling by IA.

**Figure 2:**
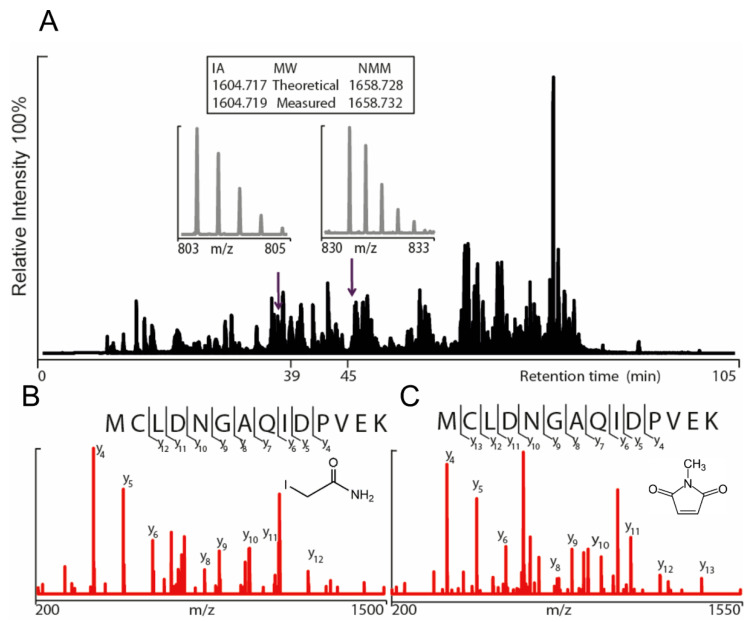
Annotation of iodoacetamide (IA) modified and *N*-methylmaleimide (NMM) modified 257–270 human TRPA1 (hTRPA1) peptides (MCLDNGAQIDPVEK) using nanoHPLC and tandem mass spectrometry. (**A**) Representative total ion chromatogram of trypsin digested hTRPA1 depicted in black. The IA and NMM modified peptides (257–270) for Cys258 were eluted at 39 min and 45 min respectively. The Orbitrap mass spectra of the double-charged Cys258-IA peptide (*m*/*z* 803.3669) and the double-charged Cys258-NMM peptide (*m*/*z* 830.3731) enabled accurate mass determination of both peptides (isotopic distribution depicted in grey). The assigned fragmentation pattern of IA (**B**) and NMM (**C**) modified peptides are given in red with indicated y-ions.

**Figure 3:**
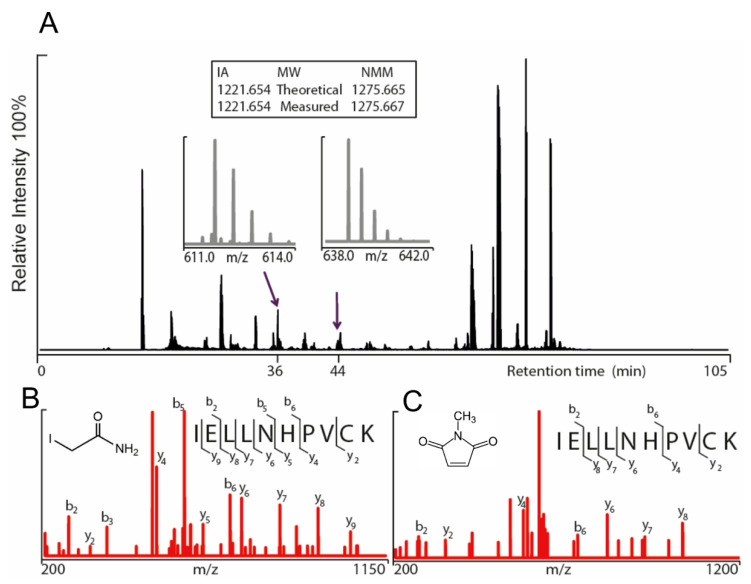
Annotation of iodoacetamide (IA) modified and *N*-methylmaleimide (NMM) modified 695–704 peptides (IELLNHPVCK) of human TRPA1 (hTRPA1) lacking the N-terminal ARD (Δ1-688 hTRPA1) using nanoHPLC and tandem mass spectrometry. (**A**) Representative total ion chromatogram of trypsin digested Δ1-688 hTRPA1 depicted in black. The IA and NMM modified peptides (695–704) for Cys703 were eluted at 36 min and 44 min respectively. The Orbitrap mass spectra of the double-charged Cys703-IA peptide (*m*/*z* 611.8342) and the double-charged Cys703-NMM peptide (*m*/*z* 638.8406) enabled accurate mass determination of both peptides (isotopic distribution depicted in grey). The assigned fragmentation pattern of IA (**B**) and NMM (**C**) modified peptides are given in red with indicated y- and b-ions.

**Figure 4:**
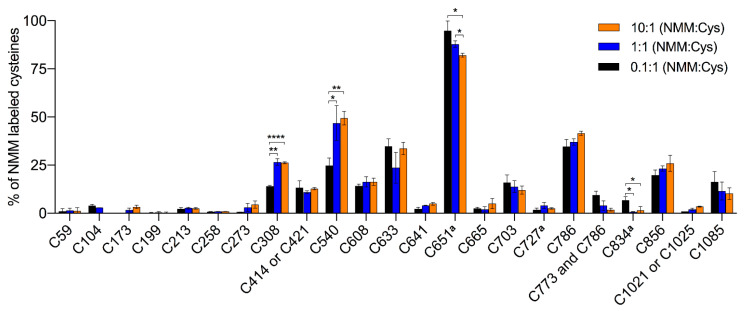
Mapping of binding sites for the electrophilic activator *N*-methylmaleimide (NMM) in human TRPA1 (hTRPA1). The bar chart shows percent site labeling of NMM. The experiments were conducted in triplicates at each concentration of NMM under a fixed reaction time. The percentage of labeling is presented as the mean with standard deviation. * *p* < 0.05, ** *p* < 0.01, and **** *p* < 0.0001 indicate that differences in labeling of cysteine at different concentrations of NMM are statistically significant using unpaired *t*-test with Welch’s correction. ^a^ Data is retrieved from the chymotrypsin digestion.

**Figure 5:**
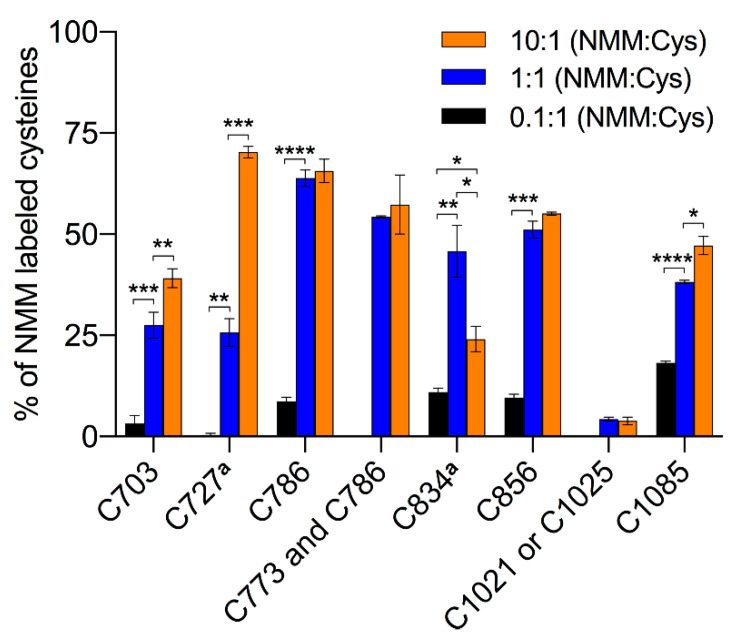
Mapping of binding sites for the electrophilic activator *N*-methylmaleimide (NMM) in human TRPA1 (hTRPA1) lacking the N-terminal ARD (Δ1-688 hTRPA1). The bar chart shows percent site labeling of NMM. The experiments were conducted in triplicates at each concentration of NMM under a fixed reaction time. The percentage of labeling is presented as the mean with standard deviation. * *p* < 0.05, ** *p* < 0.01, *** *p* < 0.001 and **** *p* < 0.0001 indicate that differences in labeling of cysteine at different concentrations of NMM are statistically significant using unpaired *t*-test with Welch’s correction. ^a^ Data is retrieved from the chymotrypsin digestion.

Two different constructs of the hTRPA1 protein, with and without the N-terminal ARD, were purified as described previously [9] with the modification that any reducing agent was completely avoided in all purification steps. In order to compare the labeling of cysteines and to probe for agonist-induced conformational changes of hTRPA1, the solubilized proteins were individually incubated with three different molar ratios of NMM in respect to the total cysteines of the protein (Figure 1). Gel electrophoresis was performed for the purpose of removal of contaminants and bands corresponding to monomeric molecular weight were excised from the gel. These gel pieces were treated with DTT to reduce any disulphide bonds and subsequently alkylated with IA to prevent reformation of disulphide bonds. Trypsin and chymotrypsin were used for the in-gel digestion of the proteins and the resulting peptide samples were submitted to LC MS/MS analysis. Both enzymatic digestions gave good sequence coverage of the two hTRPA1 proteins which allowed identification of all cysteine-containing peptides except for Cys192, which was not detected in any enzymatic digestion (Data S1). The reactivity of individual cysteines in the hTRPA1 proteins was monitored in the form of relative labeling by NMM, using two different cysteine modifying agents, NMM at the folded state and IA at a subsequent reduced and denatured state. The NMM labeling was quantified by calculating the area of the NMM modified peptide, and it was related to the sum of the areas of the NMM modified and the corresponding IA modified peptides (Figure 2 and Figure 3). The results from experiments in triplicates at each ratio of NMM to cysteines with hTRPA1 and Δ1-688 hTRPA1 are summarized in Figure 4 and Figure 5, and numeric values are listed in Table 1 and Table 2.

### 2.1. NMM Adducts to hTRPA1 Cysteines

**Figure 6:**
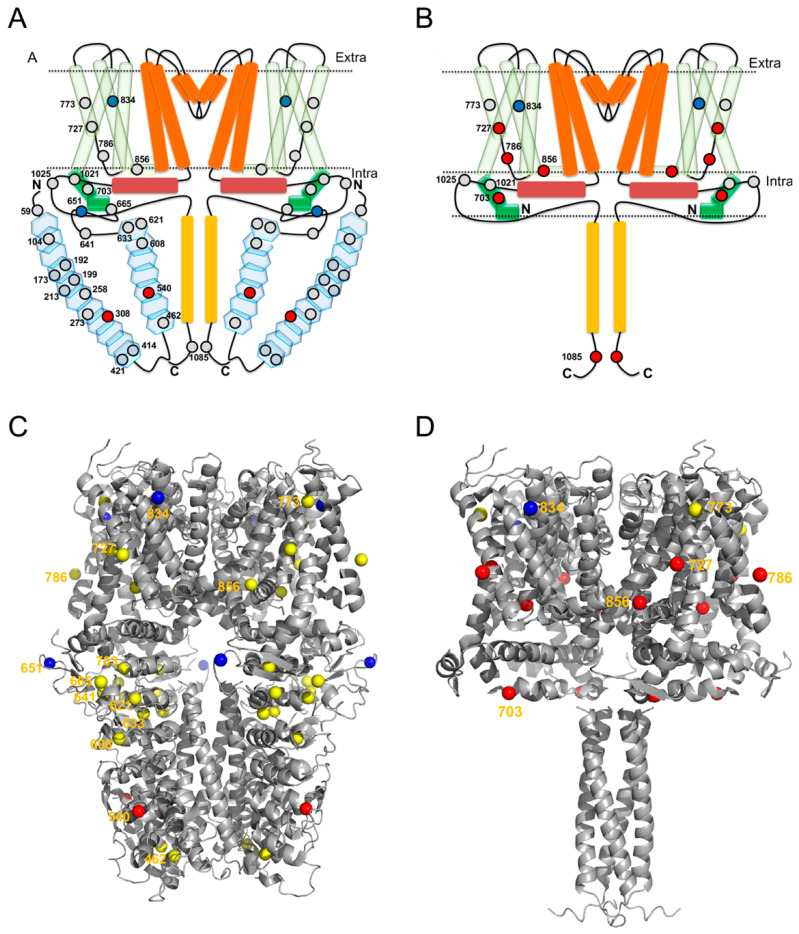
Structural localization of human TRPA1 (hTRPA1) cysteines and their reactivity to *N*-methylmaleimide (NMM). (**A**,**B**) Schematic representation of structural elements in hTRPA1 and position of cysteine residues. The subunits in the hTRPA1 are arranged into a tetramer surrounding a common ion permeation pathway. For clarity, only two subunits of the tetramer are displayed. (**A**) Each subunit of hTRPA1 contains 28 cysteines highlighted by circles. The red and blue circles are representing an increase and a decrease in NMM labeling of the cysteines, respectively, in response to increased concentrations of NMM. (**B**) The N-terminal ARD deleted version of hTRPA1 (Δ1-688 hTRPA1) has only 9 cysteines per monomer and a deviating pattern of NMM labeling. The domain organization of TRPA1 is represented in different colors, from N-terminus to C-terminus; Ankyrin repeat domain—blue (transparent), pre-S1 region—green (transparent), S1-S4 α-helices—light green (transparent), S5-S6 and pore helices—orange, TRP like domain—brick red, coiled-coil domain—yellow. (**C**,**D**) Single particle cryo-EM structure of human TRPA1 (PDB ID: 6V9W). (**C**) Cartoon representation of hTRPA1 showing a side view of the resolved part of the tetrameric assembly with the transmembrane domain at the top. 14 of the 28 cysteines in the monomer are resolved in the structure and their sulphur atoms are shown as spheres. (**D**) The same representation but with the cytosolic N-terminal ARD completely removed displaying 6 of the 9 cysteines present in the truncated monomer of hTRPA1 (Δ1-688 hTRPA1). The red and blue colored spheres represent cysteines exhibiting an increase and decrease, respectively, in the NMM labeling at higher concentrations of NMM. For clarity, the labels of the different cysteine residues are only displayed once, and in the monomer where it is best visible.

The hTRPA1 protein contains 28 cysteines, of which 20 are located in the N-terminal domain, 5 in the transmembrane domain and 3 in the C-terminal domain (Figure 6A,C). In line with a previous mass spectrometric study of mTRPA1 [20], we identified NMM adducts to all of the cysteines in the covered peptides of hTRPA1 except for Cys621, and for Cys414/Cys421 and Cys1021/Cys1025, where it was not resolved which of the two cysteines in the peptide that carried the modification (Figure 4 and Table 1). In contrast, all of the peptides containing Cys462 were labeled by NMM, but none by IA, which excluded a reliable assessment of labeling at this residue. To probe for reactivity and conformational changes in the protein, three different concentrations of NMM were used with a fixed reaction time. The lower concentrations with limiting amounts of NMM are anticipated to yield lower labeling and higher concentrations expected to increase the labeling of cysteines in the applied constant reaction time. A lower estimate of the average cysteine labeling at the 0.1 NMM:Cys ratio was 10.8 ± 0.2% (unquantified and undetected cysteines were counted as 0% labeled), which is slightly higher than a presumed maximal average labeling of 10%. At this ratio, the labeling at individual sites ranged from 0 to 95%, suggesting a huge variation in reactivity among the cysteines and specifying Cys651 as most readily labeled cysteine residue possibly with the exception of Cys462. Contrary to our expectations, a ten-fold or even a hundred-fold increase of the NMM:Cys ratio did not increase the labeling of the majority of the cysteines much, suggesting that they are largely inaccessible in the structure or unreactive due to engagement in disulphide bridges. At 13 of these sites, 90% or more of the population of hTRPA1 is unlabeled, indicating a relatively high degree of homogeneity among the subunits.

In contrast to the majority of cysteines that showed saturation of labeling already at the lowest ratio of NMM:Cys, Cys308 and Cys540 increased labeling at the approximated equimolar ratio of NMM. This concentration is sufficient to reach their maximal binding of NMM, around 25 and 50%, corresponding to an average labeling of Cys308 and Cys540 in one and two subunits, respectively, in the tetramer. Interestingly, the labeling at Cys651 showed a small but significant decrease at the highest concentration of NMM, and similarly, these higher ratios of NMM:Cys almost abolished the already poor labeling of Cys834.

### 2.2. NMM Adducts to Δ1-688 hTRPA1 Cysteines

The truncated hTRPA1 (Δ1-688 hTRPA1) construct provides a simplified model system of hTRPA1, since it contains only 9 of the 28 cysteines of hTRPA1 (Figure 6B,D) and still can be activated by electrophiles including NMM [9]. NMM adducts were observed to all cysteines except that it was not resolved which of Cys1021 and Cys1025 that was modified in their common peptide. The average labeling was 5.7 ± 0.1% at the lowest ratio, ranging to 40.3 ± 1.1% at the highest ratio. The lowest concentration of NMM results in little or no labeling, but suggests that in the truncated construct Cys1085 has the highest reactivity at limiting amounts of NMM (Figure 5 and Table 2). At an equimolar concentration of NMM an increased labeling of all cysteines was observed, and several cysteines other than Cys1085 are dominating, with Cys786 being most prominent in the labeling at this concentration. A further 10-fold increase in the NMM concentration resulted in a dramatic increase in labeling of Cys727 and a more modest but significant rise in labeling of Cys703 and Cys1085. Similar to the full length hTRPA1, the increased labeling of cysteines was accompanied by a decrease in labeling of Cys834.

### 2.3. NMM Adducts to Lysines

In addition to cysteines, NMM adducts to lysines were also observed in hTRPA1 but not quantified. The NMM modifications of Lys635, Lys704 and Lys787 in hTRPA1 were identified in reactions at all three concentrations of NMM. At higher concentrations of NMM, Lys649 was also modified. In line with this, Lys704 and Lys787 in Δ1-688 hTRPA1 were labeled by NMM at all concentrations (Table 3).

## 3. Discussion

The labeling of individual cysteines of full length hTRPA1 and a truncated version lacking the large N-terminal ARD (Δ1-688 hTRPA1), were assessed by LC MS/MS after incubations with different concentrations of NMM in a fixed period of time. The NMM labeling demonstrated a concentration-dependent modification of certain cysteines in hTRPA1, Cys308 and Cys540 showed an increase in labeling whereas Cys651 and Cys834 showed decrease in labeling with higher concentrations of NMM. A similar pattern of bidirectional labeling in response to increasing ratios of NMM to cysteines (NMM:Cys) was also seen in the truncated Δ1-688 hTRPA1 protein. Although the three first responsive cysteines of hTRPA1 (Cys308, Cys540 and Cys651) are missing in this construct, other cysteines in particular Cys727, but also Cys703 and Cys1085 increase in labeling when Cys834 decrease in labeling (comparing 1:1 to 10:1 NMM:Cys). The decrease in labeling at Cys834 appear more convincing in the truncated protein due to the overall higher labeling. However, the relative change between the different ratios of NMM is in fact larger in the full-length protein compared to the truncated version, corresponding to a 4–10 and a 2-fold decrease, respectively. The qualitatively similar response suggests that the structural changes in the two proteins are related. These findings indicate that binding of NMM to hTRPA1 and Δ1-688 hTRPA1 triggers conformational changes that make some cysteines more reactive and turn some cysteines more unreactive to further binding of NMM at higher concentrations. The reactivity of different cysteines depend both on the binding affinity i.e., the on and off rates in formation of a non-covalent NMM:TRPA1 complex, and on the rate of the reaction resulting in the covalent labeling of the thiol. At a lower concentration of NMM, the affinity is more important for the labeling whereas a higher initial concentration allows also binding at sites that are less accessible and have lower affinity. In addition to steric hindrance, also the chemical microenvironment of the cysteine in the native protein determine the reactivity of the thiol to compounds [22]. Close proximity to positively charged amino acids will lower the p*K*a value of thiols and favor their ionization, resulting in higher reactivity of the cysteines [23]. Bahia et al., reported that hTRPA1 possesses a subpopulation of highly reactive cysteines of which Cys621 shows an exceptionally high reactivity. The authors proposed that the reactivity of Cys621 is dependent on the neighboring Lys620, which likely lower the p*K*a value of Cys621 [11]. Indeed, recent cryo-EM studies support the importance of Cys621 in the activation cascade triggered by certain electrophiles [24,25]. Thus, changes in reactivity of the cysteines may be caused by rather small structural alterations at the binding sites.

A previous study on the hTRPA1 ortholog from mouse reported similar observations of decreased and increased labeling in response to a higher concentration of NMM, although the changes did not occur at the corresponding cysteines in the two orthologs. Wang et al. observed that Cys213, Cys258 and Cys727 became more accessible while Cys31, Cys273 and Cys621 turned less accessible in mTRPA1 at the higher concentration of NMM (hTRPA1 numbering of cysteines except Cys31), the authors suggested that the altered reactivity of cysteines is a result of NMM-induced conformational changes, that can lead to either channel activation or desensitization [20]. The majority of these cysteines are located in the distal N-terminal region, which displayed relatively little labeling by NMM in hTRPA1. Species dependent differences or differences in conformation due to performing the labeling at different temperatures (hTRPA1 22 °C; mTRPA1 37 °C) may explain the deviation, but an even more likely explanation is that the purified hTRPA1 is in a more oxidized state, making it difficult to monitor changes at these residues. The latter is supported by the fact that 300 µM DTT was used in the purification of mTRPA1 whereas no reducing agent was used in the preparation of hTRPA1 to provide a redox environment similar to our functional studies on purified hTRPA1 with and without the N-terminal ARD [9,26]. In fact, in the absence of reducing agents Wang et al., reported that five cysteines located in the N-terminal region are involved in the formation of four disulphide bonds, which are Cys665-Cys621, Cys665-Cys462, Cys665-Cys192 and Cys621-Cys608. When exposed to oxidants, additional disulphide bonds may appear [8] that could further change the protein conformation as well as the binding pattern of electrophilic compounds such as NMM. Although Cys621 and Cys665 may not always be the starting point of electrophile TRPA1 activation [9,15,16,17,25], these cysteines stand out as central in the formation of the TRPA1 N-terminal ARD disulphide network [8,11,20,24,25]. Indeed, in our experiments, no NMM adducts were observed to Cys621 and relatively little NMM labeling was observed to Cys665, which is consistent with their central role in a disulphide network. Thus, depending on the hTRPA1 redox state and the N-terminal ARD conformation, gating of TRPA1 by electrophiles as well as temperature and pressure may very well occur outside the N-terminal ARD [9,26,27]. Interestingly, the N-terminal ARD is not only sensitive to modifications of cysteines and lysines but also to single point mutations of other amino acids changing the mTRPA1 temperature properties from cold to heat-sensitive [28]. It is also important to take into consideration that other known intracellular modifiers of TRPA1 (e.g., pH, Ca^2+^, polyphosphates) as well as temperature, pressure and membrane potential [1,2,27,29,30], alone or in combination, may change channel conformation and the prerequisite for the N-terminal ARD in TRPA1 gating.

For some of the cysteines, the labeling at the lowest ratio was higher in hTRPA1 compared to Δ1-688 hTRPA1. This was most evident for Cys786 but also seen for Cys703 and Cys856, and could be an indication of a higher affinity for NMM at these sites in hTRPA1. Alternatively, it may be explained by the effectively higher ratio of NMM to cysteines due to the many highly inaccessible cysteines and an overestimation of the added amount of full length hTRPA1 since its lower purity compared to Δ1-688 hTRPA1 was not taken into account. At the two higher ratios the relative labeling is higher at all sites in Δ1-688 hTRPA1 (Figure 2 and Figure 3; Table 1 and Table 2). An exposure of residues at the cytosolic N- and C-terminal regions of Δ1-688 hTRPA1 can be expected if they are protected by the ARD in the full-length protein (Figure 6). However, the increased labeling of the sites deep within the transmembrane domain is more difficult to rationalize since there is no direct contact to the deleted region. A possible interpretation is that the shortened construct is overall more dynamic and allow temporal structural changes whereas the conformation of hTRPA1 is tightly controlled by the ARD and therefore more static. It is intriguing that labeling at some of the sites in the transmembrane domain like Cys786 and Cys856 reach an apparent saturation far from 100% already at NMM:Cys ratios of 0.1:1 for hTRPA1 and 1:1 for Δ1-688 hTRPA1. Although Cys856 has been proposed to be involved in disulphide bonds [7], there is no support in the structure for any of these residues forming disulphide bonds within the protein which could have provided an explanation for the limited labeling. It cannot be excluded that a change in channel conformation limits the modification by NMM also at these sites. It is tempting to speculate that it is the modification of cysteines that show an increased labeling at higher ratios of NMM:Cys that trigger the conformational switch by stabilizing the new conformation. This would indicate that although the change of conformation is similar in the transmembrane domain of hTRPA1 and Δ1-688 hTRPA1 as judged from the common decreased labeling of Cys834 at the highest ratio, the sites that induce the change are different. Consistently, the conformational switch in hTRPA1 occured at a lower NMM:Cys ratio (1:1) rather than 10:1, which is the ratio observed to change conformation of Δ1-688 hTRPA1. Importantly, NMM at 100 µM, which is within the NMM:Cys 0.1:1–1:1 ratio in the present study, activated both hTRPA1 and Δ1-688 hTRPA1 when purified and reconstituted into artificial lipid bilayers, albeit with different single-channel conductance and open probability characteristics, and with no obvious sign of desensitization [9]. Whether a higher concentration corresponding to an NMM:Cys ratio of 10:1 will cause functional desensitization of the purified hTRPA1 proteins remains, however, to be explored.

In addition to cysteines, mutations of lysines have been shown to affect the activation of TRPA1 by thiocyanates and ketoaldehydes [5,13], and targeting of lysines by electrophiles has been suggested to bypass the N-terminal ARD two step cysteine electrophile activation [25]. Thus, modifications of lysines by NMM cannot be excluded to cause the change in conformation. In general, maleimides are specific for cysteines and only react with thiols at pH 7, but at more alkaline conditions they can also interact with amines [31]. Here, we have identified that Lys649, Lys704, and Lys787 of hTRPA1 were modified by NMM at pH 7.8. Possibly a more basic pH would render also other lysines susceptible to modification by NMM. In this context, it is interesting to note that alkalization has been suggested to activate TRPA1 [32]. However, when intracellular acidosis occurs, e.g., in certain pain conditions, it seems likely that cysteines and not lysines are prime targets for electrophilic activation.

In summary, our data suggest that electrophiles induce conformational changes both in the N-terminal region and in the S1–S4 domain of hTRPA1. Our mass spectrometric analysis on Δ1-688 hTRPA1 together with functional studies [9], suggest that cysteines outside the N-terminal region are indeed involved in the detection and activation of TRPA1 by electrophiles, although this might vary depending on the chemical nature of the electrophile. In addition, several lysines in the transmembrane domain and the proximal N-terminal region were labeled by NMM, raising the possibility that lysines are also key targets for electrophilic activation of hTRPA1. Future studies combining mass spectrometry with the recent advances in cryo-EM will help to fully understand the role of the N-terminal disulphide network and the overall reactive cysteines and lysines in hTRPA1 electrophile activation.

## 4. Materials and Methods

### 4.1. Protein Expression and Purification

The expression and purification were done as described previously [9]. Two N-terminally His-tagged constructs of the hTRPA1 protein, a full length hTRPA1 and a truncated Δ1-688 hTRPA1 version, were overexpressed in the *Pichia pastoris* expression system. The cells were resuspended in cooled breaking buffer (50 mM NaH_2_PO_4_ (monobasic), 2 mM EDTA, 20% glycerol pH 7.4) containing the protease inhibitors 1 mM PMSF (1 mM), pepstatin (1 µg/mL) and leupeptin (1 µg/mL), all from Sigma-Aldrich. The resuspended cells were transferred to a Bead Beater (BioSpec Products) container having 0.5 mm cold glass beads. The cells were mechanically broken by 12 × 30 s cycles in 30 s intervening cooling sessions in a cold room (8 °C). Cell debris and unbroken cells were removed by using low-speed centrifugation 1380× *g* for 30 min. Membranes were collected by ultracentrifugation (185,000× *g* for 2 h) and homogenized in buffer A (1 × PBS, 500 mM NaCl, pH 7.8).

The membranes were solubilized in buffer A containing 2% Fos-choline-14 (Anatrace) at room temperature for 2 h with mild stirring. Detergent-insoluble membranes were removed by ultracentrifugation 100,000× *g* for 30 min. The solubilized membranes were incubated with pre-equilibrated Ni-NTA agarose resin (Qiagen) for 2 h in a cold room (8 °C). Imidazole 10 mM and 20 mM was added to prevent unspecific binding to the resin for hTRPA1 and Δ1-688 hTRPA1, respectively. The binding solution was transferred to a poly-prep chromatography column (Bio-Rad) washed with buffer B (1 × PBS, pH 7.8, 500 mM NaCl, 0.014% Fos-choline-14) containing 50 mM (hTRPA1) or 120 mM (Δ1-688 hTRPA1) imidazole. The proteins were eluted with the same buffer, containing 300 mM imidazole, after which the samples were loaded onto a pre-equilibrated Superdex 200 column (GE Healthcare), and the proteins were collected in a PBS buffer (10% glycerol, 0.014% Fos-choline-14 pH 7.8). After purification, the TRPA1 proteins were concentrated with Vivaspin 4 with a 50 kDa MWCO (Sartorius Stedim Biotech). The protein concentration was determined by measuring absorbance at 280 nm using Nano drop spectrophotometer. The extinction coefficients were estimated based on protein amino acid sequence using web tool (http://web.expasy.org/protparam/). At 280 nm the extinction coefficients for hTRPA1 and Δ1-688 hTRPA1 in water were 109,670 M^−1^cm^−1^ and 56,840 M^−1^cm^−1^, respectively.

### 4.2. Labeling with N-Methylmaleimide

To allow labeling, the metal affinity chromatography purified and desalted TRPA1 proteins were each incubated at room temperature (22 °C) for 10 min at three different molar ratios (0.1, 1, and 10) of N-methylmaleimide (NMM) in relation to cysteines present in the TRPA1 proteins. In a final volume of 14 µL 0.23 nmol of hTRPA1 containing 28 cysteines was mixed with 0.64, 6.43 or 64.26 nmol of NMM, while the corresponding amounts for 0.56 nmol of Δ1-688 hTRPA1 which has 9 cysteines were 0.51, 5.06, and 50.58 nmol of NMM. Dithiothreitol (DTT, 30 mM) was used to stop the reaction of NMM by quenching. After addition of Laemmli sample buffer (4% SDS, 20% glycerol, 10% β-mercaptoethanol, 0.004% bromophenol blue and 125 mM Tris-HCl pH 6.8), the samples were loaded on 12% TGX gels (Bio-Rad) and electrophoresis was performed.

### 4.3. In-Gel Digestions

To visualize the proteins, the SDS-polyacrylamide gels were stained with Coomassie Blue and destained with 25% methanol and 7% acetic acid in water to remove the background stain. Bands corresponding to the monomeric protein were excised and washed with distilled water and then with 50 mM ammonium bicarbonate (NH_4_HCO_3_) and 50% acetonitrile solution for 1–2 h to remove the stain from the protein. The gel pieces were dehydrated with 100% acetonitrile prior to reduction of the disulphide bonds using 10 mM DTT in 50 mM NH_4_HCO_3_ for 30 min at 37 °C. After reduction, the gel pieces were dehydrated again with 100% acetonitrile prior to alkylation. The alkylation was performed to prevent the reformation of disulphide bonds in the protein by incubating gel pieces with the alkylating agent 55 mM iodoacetamide (IA) in 50 mM NH_4_HCO_3_ for 30 min at room temperature in the dark. The gel pieces were washed (50 mM NH_4_HCO_3_ and 50% acetonitrile) and then again dehydrated (100% acetonitrile). After removal of acetonitrile, the gel pieces were allowed to dry completely. The enzymatic digestion was carried out separately for each enzyme by adding 50 μL of freshly prepared 20 ng/μL trypsin (Promega sequencing grade modified) and chymotrypsin (Roche Applied Science sequencing grade) in 50 mM NH_4_HCO_3_ in a 20:1 enzyme: protein ratio (*w*/*w*) to the samples. The samples were incubated at 37 °C for overnight and the digestion was stopped by the addition of 0.5% trifluoroacetic acid.

### 4.4. Nano LC MS/MS

All experiments were performed in triplicates for each ratio of ligand to cysteines, employing an EasyLC nanoflow HPLC interfaced with a nanoEasy spray ion source (Proxeon Biosystems, Odense, Denmark) connected to a Fusion Orbitrap mass spectrometer (Thermo Fisher Scientific). The peptides were loaded on a 2 cm PepMap column (75 μm inner diameter) packed with 3 μm C_18_ resin and the chromatographic separation was performed at 35 °C on a 25 cm EASY-Spray column (75 μm inner diameter) packed with 2 μm of C_18_ resin (Proxeon Biosystems). The nanoHPLC was operating at 300 nL/min flow rate with a gradient of 5–20% solvent B (0.1% (*v*/*v*) formic acid in acetonitrile) in solvent A (0.1% (*v*/*v*) formic acid in water) during 60 min and then 20–40% during 30 min, followed by an increase to 90% during 5 min.

A MS scan (400–1400 *m*/*z*) was recorded in the Orbitrap mass analyzer set at a resolution of 60,000 at 400 *m*/*z*, 1 × 10 automatic gain control target, and 500 ms maximum ion injection time. The MS was followed by top speed data-dependent collision-induced dissociation MS/MS scans on multiply charged ions. The general mass spectrometric conditions were as follows: spray voltage, 1.9 kV; no sheath or auxiliary gas flow; S-lens 60%; ion transfer tube temperature, 275 °C. Collision induced dissociation was applied with 35% of energy and MS/MS spectra were recorded in the Orbitrap at a resolution of 15,000.

### 4.5. Database Search

Raw data were processed by Mascot distiller searching the Swiss-Prot database with an in-house Mascot database. The search parameters for the Mascot searches were: Taxonomy: *Homo sapiens*; Enzymes; trypsin with one missed cleavage, chymotrypsin with five missed cleavages, Variable Modifications; IA (carbamidomethylation of Cys), NMM (Cys and Lys), and oxidation (Met), Precursor Tolerance, 5 ppm; MS/MS Fragment Tolerance, 0.1 Da. The modifications of detected peptides were identified by adding a mass of 111.0320 Da for NMM and 57.0516 Da for IA to the cysteines in the database search. The peaks corresponding to IA and NMM modified peptides in MS mode were extracted and peak areas were calculated using the Xcalibur Software (Thermo Fisher Scientific).

## Figures and Tables

**Table 1:** The percentage of *N*-methylmaleimide (NMM) labeling for each cysteine in the human TRPA1 was analyzed from tryptic digested peptides. The NMM labeling was calculated for each cysteine by area of NMM modified peptide divided with total areas of NMM modified peptide and equivalent iodoacetamide (IA) modified peptide. The mass spectrometry experiments were performed in triplicates for each concentration, and the quantified data presented as the mean value with standard deviation (SD). The proteins were incubated at different concentrations of NMM, corresponding to different molar ratios relative the total number of cysteines.

Cysteine	Peptide Sequence	Range	0.1:1 NMM:Cys % ± SD	1:1 NMM:Cys % ± SD	10:1 NMM:Cys % ± SD
C59	C_NMM_DDMDTFFLHYAAAEGQIELMEK	59–81	0.98 ± 1.30	1.34 ± 1.19	1.09 ± 1.83
C104	DSSLEVLHEM_ox_DDYGNTPLHC_NMM_AVEK	85–108	3.84 ± 0.71	2.85 ± 0.04	0.04 ± 0.00
C173	TIDVNLEGENGNTAVIIAC_NMM_TTNNSEALQILLK	155–186	0.05 ± 0.07	1.65 ± 1.05	3.32 ± 0.92
C199	WGC_NMM_FPIHQAAFSGSK	197–211	0.30 ± 0.13	0.42 ± 0.34	0.25 ± 0.25
C213	EC_NMM_M_ox_EIILR	212–219	2.21 ± 0.74	2.68 ± 0.47	2.52 ± 0.40
C258	M_ox_C_NMM_LDNGAQIDPVEK	257–270	0.76 ± 0.12	0.87 ± 0.10	0.87 ± 0.10
C273	C_NMM_TAIHFAATQGATEIVK	273–289	0.61 ± 0.04	3.00 ± 2.02	4.53 ± 1.87
C308	LM_ox_ISSYSGSVDIVNTTDGC_NMM_HETMLHR	290–315	14.01 ± 0.45	26.56 ± 1.71	26.18 ± 0.49
C414 or C421	ELVM_ox_DEDNDGC_IA_TPLHYAC_NMM_R	404–422	13.28 ± 3.47	10.88 ± 0.71	12.80 ± 0.57
C462 ^b^	INTC_NMM_QR	459–464	all	all	all
C540	C_NMM_TDRLDEDGNTALHFAAR	540–557	24.68 ± 4.02	46.81 ± 9.05	49.38 ± 3.58
C608	WDEC_NMM_LK	605–610	14.15 ± 0.86	16.31 ± 2.61	16.28 ± 1.89
C633	C_IA_PITEM_ox_IEYLPEC_NMM_M_ox_K	621–635	34.73 ± 3.88	23.65 ± 7.99	33.62 ± 3.17
C641	VLLDFC_NMM_M_ox_LHSTEDK	636–649	2.25 ± 0.84	4.05 ± 0.16	4.95 ± 0.71
C651 ^a^	HSTEDKSC_NMM_RDY	644–654	94.78 ± 5.02	87.83 ± 1.68	82.04 ± 0.98
C665	YLQC_NMM_PLEFTK	662–671	2.52 ± 0.63	1.98 ± 1.39	4.96 ± 2.62
C703	IELLNHPVC_NMM_K	695–704	15.90 ± 3.96	13.76 ± 3.13	12.04 ± 2.11
C727 ^a^	C_NMM_LGLIPM_ox_TIL	727–736	1.76 ± 0.94	3.99 ± 1.52	2.52 ± 0.40
C773 and C786	TC_NMM_M_ox_ILVFLSSIFGYC_NMM_K	772–787	9.38 ± 2.11	3.96 ± 2.52	1.82 ± 0.80
C786	TC_IA_M_ox_ILVFLSSIFGYC_NMM_K	772–787	34.59 ± 3.70	36.94 ± 1.68	41.47 ± 1.05
C834 ^a^	QWQC_NMM_GAIAVY	831–840	6.73 ± 1.83	0.66 ± 0.12	1.53 ± 1.97
C856	FENC_NMM_GIFIVMLEVILK	853–868	19.81 ± 2.62	23.16 ± 1.40	25.89 ± 4.16
C1021 or C1025	SGGM_ox_LFHIFC_IA_FLFC_NMM_TGEIR	1012–1030	0.87 ± 0.03	1.99 ± 0.53	3.44 ± 0.24
C1085	M_ox_EIISETEDDDSHC_NMM_SFQDR	1072–1090	16.27 ± 5.38	11.50 ± 4.68	10.18 ± 3.05
Average NMM labeling (lower estimate)			10.84 ± 0.19	11.27 ± 0.43	11.79 ± 0.38

^a^ Data is retrieved from chymotrypsin digestion. ^b^ All detected peptides by MS were modified by NMM. In the calculation of average NMM labeling, all the NMM modified cysteines are taken into account and those not modified by NMM or not identified in the sequence are counted as zero value.

**Table 2:** The percentage of *N*-methylmaleimide (NMM) labeling for each cysteine in the Δ1-688 human TRPA1 was analyzed from tryptic digested peptides. The NMM labeling was calculated for each cysteine by area of NMM modified peptide divided with total areas of NMM modified peptide and equivalent iodoacetamide (IA) modified peptide. The mass spectrometry experiments were performed in triplicates for each concentration, and the quantified data presented in the mean value with standard deviation (SD). The proteins were incubated at different concentrations of NMM, corresponding to different molar ratios relative the total number of cysteines.

Cysteine	Peptide Sequence	Range	0.1:1 NMM:Cys % ± SD	1:1 NMM:Cys % ± SD	10:1 NMM:Cys % ± SD
C703	IELLNHPVC_NMM_K	695–704	3.21 ± 1.96	27.53 ± 3.21	39.14 ± 2.36
C727 ^a^	C_NMM_LGLIPM_ox_TIL	727–736	0.37 ± 0.40	25.70 ± 3.14	70.30 ± 1.44
C773 and C786	TC_NMM_M_ox_ILVFLSSIFGYC_NMM_K	772–787	0	54.29 ± 0.23	57.31 ± 7.29
C786	TC_IA_M_ox_ILVFLSSIFGYC_NMM_K	772–787	8.68 ± 1.06	63.89 ± 2.02	65.67 ± 2.85
C834 ^a^	QWQC_NMM_GAIAVYF	831–841	10.94 ± 0.98	45.81 ± 6.43	24.07 ± 3.12
C856	FENC_NMM_GIFIVMLEVILK	853–868	9.65 ± 0.80	51.12 ± 2.06	55.09 ± 0.38
C1021 or C1025	SGGM_ox_LFHIFC_IA_FLFC_NMM_TGEIR	1012–1030	0	4.27 ± 0.48	3.87 ± 0.96
C1085	M_ox_EIISETEDDDSHC_NMM_SFQDR	1072–1090	18.22 ± 0.38	38.19 ± 0.40	47.19 ± 2.22
Average NMM labeling (lower estimate)			5.67 ± 0.11	34.53 ± 0.24	40.29 ± 1.09

^a^ Data is retrieved from chymotrypsin digestion. In the calculation of average NMM labeling, all the NMM modified cysteines are taken into account and those not modified by NMM are counted as zero value.

**Table 3:** The observed *N*-methylmaleimide (NMM) adducts to lysines in human TRPA1 and Δ1-688 human TRPA1 from both trypsin and chymotrypsin digested peptides. The proteins were incubated at different concentrations of NMM, corresponding to different molar ratios relative the total number of cysteines.

Lysine	0.1:1 (NMM:Cys)	1:1 (NMM:Cys)	10:1 (NMM:Cys)
K635	●	●	●
K649	○	○	●
K704	●■	●■	●■
K787	●■	●■	●■

● Identified in human TRPA1. ○ Not identified in human TRPA1. ■ Identified in Δ1-688 human TRPA1.

## Supplementary Materials

The following are available online at https://www.mdpi.com/1422-0067/21/18/6667/s1, Data S1. The obtained sequence coverage of human TRPA1 and Δ1-688 human TRPA1 by mass spectrometry in trypsin or chymotrypsin digestions.
