# Supplementary material for: Electrophile-Induced Conformational Switch of the Human TRPA1 Ion Channel Detected by Mass Spectrometry
Source: Int J Mol Sci. 2020 Sep 11;21(18):6667. doi: 10.3390/ijms21186667 (PMC7555621; doi:10.3390/ijms21186667)
Supplement: Supplementary file 1 [file ijms-21-06667-s001.pdf]

Data S1. The obtained sequence coverage of human TRPA1 and Δ1-688 human TRPA1 by mass spectrometry in trypsin or chymotrypsin digestions. The red colored residues were detected in peptides resulting from the digestion with the specified enzyme, whereas black colored residues were not detected.

hTRPA1 sequence coverage of 76.2% by trypsin digestion

```
1      MKRSLRKMWRPGEKKEPQGVVYEDVPDDTEDFKESLKVVFEGSAYGLQNFNKQKKLKRCDDMDTFFLHYAAAEGQIELMEKITRDSSLEVLHEMDDYGNT
101    PLHCAVEKNQIESVKFLLSRGANPNLRNFNMMAPLHIAVQGMNNEVMKVLLEHRTIDVNLEGENGNTAVIIACTTNNSEALQILLKKGAKPCKSNKWGCF
201    PIHQAAFSGSKECMEIILRFGEEHGYSRQLHINFMNNGKATPLHLAVQNGDLEMIKMCLDNGAQIDPVEKGRCTAIHFAATQGATEIVKLMISSYSGSVD
301    IVNTTDGCHETMLHRASLFDHHELADYLISVGADINKIDSEGRSPLILATASASWNIVNLLLSKGAQVDIKDNFGRNFLHLTVQQPYGLKNLRPEFMQMQ
401    QIKELVMDEDNDGCTPLHYACRQGGPGSVNNLLGFNVSIHSKSKDKKSPLHFAASYGRINTCQRLLQDISDTRLLNEGDLHGMTPLHLAAKNGHDKVVQL
501    LLKKGALFLSDHNGWTALHHASMGGYTQTMKVILDTNLKCTDRLDEDGNTALHFAAREGHAKAVALLLSHNADIVLNKQQASFLHLALHNKRKEVVLTII
601    RSKRWDECLKIFSHNSPGNKCPITEMIEYLPECMKVLLDFCMLHSTEDKSCRDYYIEYNFKYLQCPLEFTKKTPTQDVIYEPLTALNAMVQNNRIELLNH
701    PVCKEYLLMKWLAYGFRAHMMNLGSYCLGLIPMTILVVNIKPGMAFNSTGIINETSDHSEILDTTNSYLIKTCMILVFLSSIFGYCKEAGQIFQQKRNYF
801    MDISNVLEWIIYTTGIIFVLPLFVEIPAHLQWQCGAIAVYFYWMNFLLYLQRFENCGIFIVMLEVILKTLLRSTVVFIFLLLAFGLSFYILLNLQDPFSS
901    PLLSIIQTFSMMLGDINYRESFLEPYLRNELAHPVLSFAQLVSFTIFVPIVLMNLLIGLAVGDIAEVQKHASLKRIAMQVELHTSLEKKLPLWFLRKVDQ
1001   KSTIVYPNKPRSGGMLFHIFCFLFCTGEIRQEIPNADKSLEMEILKQKYRLKDLTFLLEKQHELIKLIIQKMEIISETEDDDSHCSFQDRFKKEQMEQRN
1101   SRWNTVLRAVKAKTHHLEP
```

hTRPA1 sequence coverage of 87.8% by chymotrypsin digestion

```
1      MKRSLRKMWRPGEKKEPQGVVYEDVPDDTEDFKESLKVVFEGSAYGLQNFNKQKKLKRCDDMDTFFLHYAAAEGQIELMEKITRDSSLEVLHEMDDYGNT
101    PLHCAVEKNQIESVKFLLSRGANPNLRNFNMMAPLHIAVQGMNNEVMKVLLEHRTIDVNLEGENGNTAVIIACTTNNSEALQILLKKGAKPCKSNKWGCF
201    PIHQAAFSGSKECMEIILRFGEEHGYSRQLHINFMNNGKATPLHLAVQNGDLEMIKMCLDNGAQIDPVEKGRCTAIHFAATQGATEIVKLMISSYSGSVD
301    IVNTTDGCHETMLHRASLFDHHELADYLISVGADINKIDSEGRSPLILATASASWNIVNLLLSKGAQVDIKDNFGRNFLHLTVQQPYGLKNLRPEFMQMQ
401    QIKELVMDEDNDGCTPLHYACRQGGPGSVNNLLGFNVSIHSKSKDKKSPLHFAASYGRINTCQRLLQDISDTRLLNEGDLHGMTPLHLAAKNGHDKVVQL
501    LLKKGALFLSDHNGWTALHHASMGGYTQTMKVILDTNLKCTDRLDEDGNTALHFAAREGHAKAVALLLSHNADIVLNKQQASFLHLALHNKRKEVVLTII
601    RSKRWDECLKIFSHNSPGNKCPITEMIEYLPECMKVLLDFCMLHSTEDKSCRDYYIEYNFKYLQCPLEFTKKTPTQDVIYEPLTALNAMVQNNRIELLNH
701    PVCKEYLLMKWLAYGFRAHMMNLGSYCLGLIPMTILVVNIKPGMAFNSTGIINETSDHSEILDTTNSYLIKTCMILVFLSSIFGYCKEAGQIFQQKRNYF
801    MDISNVLEWIIYTTGIIFVLPLFVEIPAHLQWQCGAIAVYFYWMNFLLYLQRFENCGIFIVMLEVILKTLLRSTVVFIFLLLAFGLSFYILLNLQDPFSS
901    PLLSIIQTFSMMLGDINYRESFLEPYLRNELAHPVLSFAQLVSFTIFVPIVLMNLLIGLAVGDIAEVQKHASLKRIAMQVELHTSLEKKLPLWFLRKVDQ
1001   KSTIVYPNKPRSGGMLFHIFCFLFCTGEIRQEIPNADKSLEMEILKQKYRLKDLTFLLEKQHELIKLIIQKMEIISETEDDDSHCSFQDRFKKEQMEQRN
1101   SRWNTVLRAVKAKTHHLEP
```

Δ1-688 hTRPA1 sequence coverage of 52.9% by trypsin digestion

```
689                                                                                            MVQNNRIELLNH
701    PVCKEYLLMKWLAYGFRAHMMNLGSYCLGLIPMTILVVNIKPGMAFNSTGIINETSDHSEILDTTNSYLIKTCMILVFLSSIFGYCKEAGQIFQQKRNYF
801    MDISNVLEWIIYTTGIIFVLPLFVEIPAHLQWQCGAIAVYFYWMNFLLYLQRFENCGIFIVMLEVILKTLLRSTVVFIFLLLAFGLSFYILLNLQDPFSS
901    PLLSIIQTFSMMLGDINYRESFLEPYLRNELAHPVLSFAQLVSFTIFVPIVLMNLLIGLAVGDIAEVQKHASLKRIAMQVELHTSLEKKLPLWFLRKVDQ
1001   KSTIVYPNKPRSGGMLFHIFCFLFCTGEIRQEIPNADKSLEMEILKQKYRLKDLTFLLEKQHELIKLIIQKMEIISETEDDDSHCSFQDRFKKEQMEQRN
1101   SRWNTVLRAVKAKTHHLEP
```

Δ1-688 hTRPA1 sequence coverage of 87.5% by chymotrypsin digestion

```
689                                                                                            MVQNNRIELLNH
701    PVCKEYLLMKWLAYGFRAHMMNLGSYCLGLIPMTILVVNIKPGMAFNSTGIINETSDHSEILDTTNSYLIKTCMILVFLSSIFGYCKEAGQIFQQKRNYF
801    MDISNVLEWIIYTTGIIFVLPLFVEIPAHLQWQCGAIAVYFYWMNFLLYLQRFENCGIFIVMLEVILKTLLRSTVVFIFLLLAFGLSFYILLNLQDPFSS
901    PLLSIIQTFSMMLGDINYRESFLEPYLRNELAHPVLSFAQLVSFTIFVPIVLMNLLIGLAVGDIAEVQKHASLKRIAMQVELHTSLEKKLPLWFLRKVDQ
1001   KSTIVYPNKPRSGGMLFHIFCFLFCTGEIRQEIPNADKSLEMEILKQKYRLKDLTFLLEKQHELIKLIIQKMEIISETEDDDSHCSFQDRFKKEQMEQRN
1101   SRWNTVLRAVKAKTHHLEP
```
